# Interleukin-13, but Not Indomethacin, Increases Cysteinyl-Leukotriene Synthesis in Human Lung Macrophages

**DOI:** 10.1155/2012/348741

**Published:** 2011-10-29

**Authors:** Sarah E. Jackson, John W. Holloway, Jane A. Warner, Anthony P. Sampson

**Affiliations:** Infection, Inflammation and Immunity Division, University of Southampton School of Medicine, Southampton SO16 6YD, UK

## Abstract

Aspirin-exacerbated respiratory disease (AERD) is associated with constitutively elevated synthesis of bronchoconstrictor cysteinyl-leukotrienes, associated with increased expression of leukotriene (LT)C_4_ synthase and Th2 cytokines and airway eosinophilia. We examined whether interleukin-13 can increase LTC_4_ synthase gene transcription and cysteinyl-leukotriene synthesis in macrophages isolated from resected human lung tissue and whether an NSAID (indomethacin) can trigger further cysteinyl-leukotriene synthesis in these cells. Overnight culture of human lung macrophages with IL-13 (10 ng/mL) increased spontaneous and ionophore-stimulated production of cysteinyl-leukotrienes by 42% (*P* = 0.02) and 52% (*P* = 0.005), respectively, as quantified by enzyme immunoassays, but PCR gene transcription assays did not demonstrate an effect on LTC4S mRNA. The addition of indomethacin (100 *μ*M) did not modulate cysteinyl-leukotriene production in either IL-13-treated or untreated macrophages. We conclude that while IL-13 enhances cysteinyl-leukotriene synthesis in human lung macrophages, it does not replicate the enhanced LTC_4_ synthase expression observed in the AERD lung nor confer sensitivity to NSAIDs.

## 1. Introduction

Aspirin-exacerbated respiratory disease (AERD) is a syndrome in which chronic asthma is accompanied by nonallergic hypersensitivity to nonsteroidal anti-inflammatory drugs (NSAIDs), leading to acute bronchoconstriction and exacerbation of other lower and upper airway symptoms [1, 2]. The ability of classical NSAIDs to inhibit prostaglandin synthesis by cyclooxygenase (COX) isozymes, particularly COX-1, is implicated in these acute exacerbations [3], but it is not known how they activate mast cells, eosinophils, macrophages, epithelium, and other cells to release a range of inflammatory mediators in susceptible subjects. 

Prominent among these mediators are the cysteinyl-leukotrienes (cys-LTs), which are potent bronchoconstrictor lipids synthesised by the 5-lipoxygenase (5-LO)/LTC_4_ synthase pathway [4]. Compared to NSAID-tolerant asthmatics, AERD patients have chronically elevated production of cys-LTs, as demonstrated in bronchoalveolar lavage (BAL) fluid, induced sputum, exhaled breath condensate, and urine [2]. Together with the clinical efficacy of antileukotriene drugs [5], this suggests a key role of cys-LTs in chronic AERD, even when NSAIDs are entirely avoided. Exposure to NSAIDs is postulated to shunt the shared substrate arachidonic acid from the prostaglandin (cyclooxygenase) pathway to the leukotriene (5-lipoxygenase) pathway, or to reduce production of a prostaglandin, putatively PGE_2_, that normally suppresses leukotriene synthesis via an EP receptor mechanism [2], possibly via phosphorylation of LTC_4_ synthase [6]. Increased cys-LT production is prominent in the acute bronchoconstriction that results, but it is not understood why NSAIDs trigger the acute surge in cys-LT levels only in AERD subjects.

In 1998, we described with our collaborators a marked overexpression of LTC_4_ synthase, the terminal enzyme for the cellular biosynthesis of the first of the cys-LTs, LTC_4_, in the bronchial mucosa of AERD patients [7]. LTC_4_ synthase overexpression was also described in nasal polyps from aspirin-sensitive rhinitic patients [8]. A model for NSAID sensitivity was proposed in which enhanced expression of LTC_4_ synthase in airway macrophages, mast cells, and eosinophils provides the enzymatic capacity for constitutive overproduction of cys-LTs in AERD [7, 9]. Indeed, total numbers of LTC_4_ synthase-positive cells in the bronchial mucosa correlate strikingly with increased cys-LT levels in BAL fluid in AERD subjects [7]. Overexpression of LTC_4_ synthase may also explain why only AERD subjects respond adversely to NSAID challenge, as suggested by the unique relationship between bronchial LTC_4_ synthase and airway hyperresponsiveness to aspirin challenge [7]. 

The view that LTC_4_ synthase overexpression is a central anomaly in AERD is supported by recent work in LTC_4_ synthase transgenic mice [10], although the potential mechanisms involved remain unclear. LTC_4_ synthase can be upregulated by cytokines, including interleukin (IL)-3 and IL-5 in maturing eosinophils [11] and IL-4 and IL-13 in human cord-blood mast cells [12], while TNF*α* downregulates LTC_4_ synthase in monocytes [13]. Immunoexpression of IL-5, but not IL-3 or GMCSF, is increased in AERD biopsies relative to aspirin-tolerant subjects [7], but there are no studies of IL-13 in AERD lung. Macrophages from resected human lung may represent a useful cellular model for AERD as they are primary cells that express both the 5-LO/LTC_4_ synthase and COX biosynthetic pathway enzymes, and also receptors for IL-13 that mediate increased expression of the CysLT_1_ receptor [14]. We hypothesised that culture of human lung macrophages with IL-13 would increase LTC_4_ synthase gene transcription and cys-LT synthesis compared to control macrophages. We also hypothesised that the NSAID, indomethacin, would trigger a further release of cys-LTs only in the IL-13-treated cells.

## 2. Methods

### 2.1. Materials

RNALater and the DNA Mastermix were purchased from Ambion (Warrington, UK). TaqMan Universal Master Mix, *β*-actin and LTC4S gene expression assays, and MicroAmp optical adhesive film were purchased from Applied Biosystems (Warrington, UK). The ImProm-II reverse transcription system was purchased from Promega (Southampton, UK). The cysteinyl-leukotriene enzyme immunoassay (EIA) kit was from Cayman Chemical Europe (Tallinn, Estonia). TRIzol was purchased from Invitrogen (Paisley, UK). Recombinant human IL-13 was purchased from PeproTech (London, UK). Calcium ionophore calcimycin (A23187), indomethacin, Trypan Blue solution (0.4%), and dimethylsulphoxide (DMSO) were from Sigma Chemical Company (Poole, UK).

### 2.2. Isolation and Culture of Human Lung Macrophages

Samples of lung tissue (wet weight 1.8–35.2 g) were collected from male and female patients undergoing bullectomy or lobectomy for lung cancer at the Southampton General Hospital, in accordance with ethical approval (08/HO502/32) from the Southampton and South-West Hampshire Research Ethics Committee. None had a history of asthma, but all were current or exsmokers. Tissue samples were dissected into 3 mm fragments and suspended in Dulbecco's phosphate-buffered saline (PBS) containing 0.1 M NaCl, 2.7 mM KCl, 1.8 mM KH_2_PO_4_, and 10 mM Na_2_PO_4_ (pH 7.4). The suspension was centrifuged (80 ×g, 20°C) for 5 min, the supernatant was discarded, and the pellet was resuspended in 30 mL of lysis buffer (155 mM NH_4_Cl, 10 mM KHCO_3_, 0.1 mM EDTA; pH 7.4). After 5 min at room temperature, the suspension was filtered (70 *μ*m filter) and centrifuged (80 ×g, 20°C, 5 min) to remove erythrocyte fragments and other debris. The pellet was resuspended in RPMI medium supplemented with antibiotics (penicillin 55 U/mL, streptomycin 5 mg/mL, and gentamycin 10 mg/mL). The resulting cell population was >90% pure macrophages by morphology and had viability >90% as determined by exclusion of Trypan Blue dye. Macrophage aliquots were cultured in RPMI (0.5 × 10^6^ cells/mL) at 37°C for 16 h overnight with or without IL-13 (10 ng/mL) in a 5% CO_2_ humidified atmosphere. 

For cys-LT assays, IL-13-treated and untreated cells were incubated for a further 30 min in fresh RPMI medium (0.5 × 10^6^ cells in 1 mL) in a 37°C water bath with no addition (control), with indomethacin (100 *μ*M), with calcimycin (A23187, 1 *μ*M), or with both indomethacin and A23187 at the same concentrations. Calcimycin (A23187) is a calcium ionophore that liberates arachidonic acid from membrane phospholipids and activates 5-LO to initiate leukotriene synthesis. Indomethacin is a classical NSAID which inhibits both COX-1 and COX-2 at the concentration used. These reagents were diluted from stock solutions in dimethylsulfoxide (DMSO) such that final DMSO concentration in the cell incubations was always <0.2%. At the end of the incubation, tubes were removed onto ice and centrifuged (240 ×g, 4°C) for 5 minutes to pellet the cells for RNA analyses. The supernatants were treated with two volumes of ethanol to precipitate protein, which was removed by centrifugation. Ethanolic supernatants were then evaporated to dryness *in vacuo* in a GyroVap rotary evaporator and stored at −20°C before cys-LT immunoassays. 

### 2.3. RT-qPCR Assay for LTC4S

For LTC4S gene transcription assays, aliquots (10 × 10^6^ cells) of IL-13-treated and untreated cell pellets were mixed with 0.5 mL RNALater and kept at 4°C for 24 h, then stored at −20°C before RNA extraction and reverse-transcriptase quantitative polymerase chain reaction (RT-qPCR) assays for LTC4S and beta-actin mRNAs. Macrophages were thawed, vortexed, and transferred into RNA-free Eppendorf tubes. PBS (1 mL) was added, and Eppendorfs were vortexed and centrifuged (2800 ×g, 20°C) for 5 min to decrease the viscosity of the RNALater and allow the macrophages to form a pellet. RNALater was then removed and the RNA extracted using TRIzol (Invitrogen, Paisley, UK) by the manufacturer's protocol. Residual genomic DNA was digested using Ambion DNA-*free* (Applied Biosystems, Warrington, UK). For each RNA sample, the A260/A280 ratio measured by spectrophotometry (Nanodrop ND1000, ThermoFisher Hemel Hempstead, UK) was >1.8, indicating an adequate level of purity, and RNA was stored at −80°C. To generate cDNA, 1 *μ*g of RNA was reverse transcribed (Improm-II RT system, Promega, Southampton, UK) using random hexamer primers and stored at −20°C. 

RT-qPCR was performed on the Lightcycler 480 (Roche Diagnostics, UK) in 384-well reaction plates using ~100 ng of template cDNA in quadruplicate 20 *μ*L reactions using TaqMan gene expression assays for LTC4S (Hs00168529_m1) with ACTB (beta-actin, Hs99999903_m1) as a reference gene, both using FAM-labelled hydrolysis probes and TaqMan universal mastermix II for 40 cycles, as in the manufacturer's protocol (Applied Biosystems). Gene expression relative to ACTB was calculated using the 2^−ΔΔCq^ method. Paired statistical comparison between IL-13-treated and untreated cells from *n* = 12 donors was performed by Wilcoxon signed rank test for nonparametric data.

### 2.4. Cysteinyl-Leukotriene Immunoassays

Evaporated supernatants from 30 min incubations of IL-13-treated and untreated macrophages with and without indomethacin and calcimycin were resuspended in appropriate volumes of PBS buffer and aliquots taken in duplicate for EIA quantification of released cys-LTs. The total cys-LT EIA kits (Cayman Europe) use a monoclonal primary antibody with 100% specificity for LTC_4_ and LTD_4_ and 79% specificity for LTE_4_. Cross-reactivity to LTB_4_, various HETEs, and arachidonate is less than 4% and the assay has high sensitivity (34 pg/mL) for cys-LTs. The assay is based on competition with a standard LTC_4_-acetylcholinesterace tracer with Ellman's reagent as substrate. Cys-LTs were assayed in duplicate, and concentrations are expressed as nanograms of LTC_4_ released per million viable macrophages. Data are presented as mean ± SEM for *n* = 8 tissue donors, and comparisons between mean values were made by two-tailed paired Student's *t*-tests, with *P* < 0.05 considered significant. 

## 3. Results

**Figure 1:**
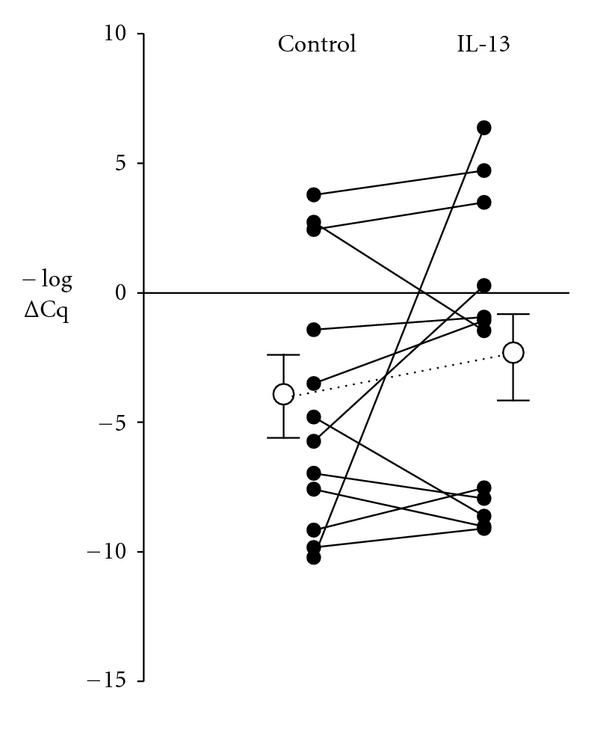
RT-qPCR assay data for LTC4S mRNA in macrophages from 12 lung tissue donors after 16 hours of culture in the presence and absence of 10 ng/mL IL-13. Values are normalised to the housekeeping gene (*β*-actin), with ΔCq values representing the number of doubling cycles taken to reach the threshold, and plotted on an inverted logarithmic *y*-axis (as −ΔCq) so that an increase in LTC4S mRNA is shown as a higher value on the axis. The mean log change in −ΔCq value was 0.49, representing no significant change in LTC4S mRNA with IL-13 culture (*P* = 0.3).

For LTC4S gene transcription assays, human lung macrophages from 12 donors (9 male, three female; mean age 64 years, range 49–78 years) were cultured overnight for 16 hours with or without IL-13 (10 ng/mL). LTC4S mRNA expression in each cell sample was detected in quadruplicate using RT-qPCR, with *β*-actin as the housekeeping gene. The mean expression of LTC4S mRNA at 16 hours was not significantly different in IL-13-treated macrophages compared to their untreated cells (mean log⁡ ΔΔCq value = 0.49, *P* = 0.33, *n* = 12) (Figure 1). The comparison remained nonsignificant when the single outlying value, caused by an anomalously low control (*β*-actin) value in the untreated cells from one donor, which was not apparent in the IL-13-treated cells from the same donor, was excluded from the analysis. 

**Figure 2:**
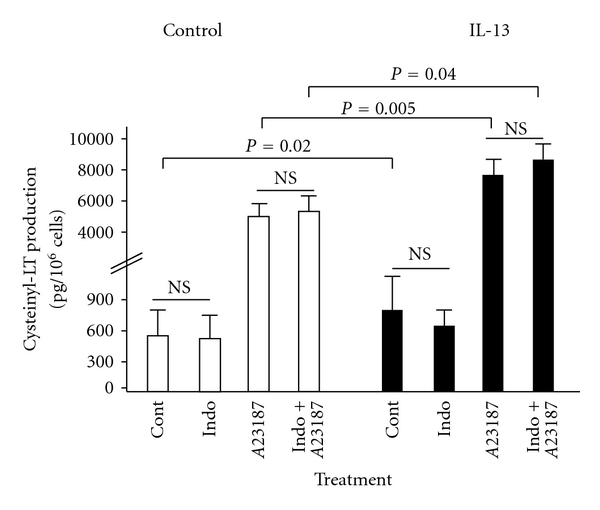
Enzyme immunoassay data on release of total cysteinyl-leukotrienes (cys-LT, pg/million cells) by macrophages from eight lung tissue donors after culture for 16 hours in the absence or presence of IL-13 (10 ng/mL). Macrophages were then washed and resuspended in fresh medium for a 30 min incubation with no further addition (Cont), with the NSAID indomethacin (Indo, 100 *μ*M), with the calcium ionophore calcimycin (A23187, 1 *μ*M), or with both indomethacin and calcimycin (Indo + A23187). Pretreatment with IL-13 significantly increased spontaneous cys-LT release (*P* = 0.02) and that induced by calcimycin (*P* = 0.005), but neither was affected by indomethacin in either IL-13 treated or untreated cells.

Following 16-hour cultures with or without IL-13, macrophages from representative donors (8 males, mean age 67 years, range 56–76) underwent 30-minute incubations for detection of total cys-LT release (Figure 2). IL-13 pretreatment significantly increased spontaneous cys-LT release from 544 ± 215 pg/million cells to 825 ± 292 pg/million cells (*P* = 0.02), a mean increase of 52 ± 17%. Incubation with indomethacin (100 *μ*M) did not significantly change cys-LT release when compared with spontaneous release in either IL-13-treated or untreated cells (*P* > 0.05). 

As expected, the calcium ionophore A23187 (calcimycin, 1uM) boosted the mean release of total cys-LTs by about 10-fold compared with spontaneous release. Mean release of total cys-LTs after 30 min of A23187 stimulation was significantly greater in IL-13-treated cells (7770 ± 630 pg/million cells) than in cells not treated with IL-13 (5480 ± 670 pg/million cells) (*P* = 0.005), a mean increase of 42 ± 10% (Figure 2). Coincubation of indomethacin (100 *μ*M) and A23187 (1 *μ*M) however did not show different values for cys-LT release compared with A23187 alone in either IL-13 pretreated or untreated cells.

## 4. Discussion

Interleukin (IL)-13 is a Th2 cytokine with well-established roles in promoting airway responsiveness, mucus secretion, and chemokine production in the allergic lung, acting principally via a receptor shared with IL-4 and leading to phosphorylation of the transcription factor STAT6 [15]. IL-4 can powerfully upregulate LTC_4_ synthase expression and activity in human cord-blood mast cells [12], while IL-13 can upregulate the principal receptor for cysteinyl-leukotrienes, CysLT1R, on human airway smooth muscle cells [16] and on macrophages [14]. This suggested that increased IL-4/IL-13 activity may be responsible for upregulating tissue expression of LTC_4_ synthase, as observed in the upper and lower airways in AERD patients [7, 8], and for enhancing responsiveness to cys-LTs by increasing the expression of CysLT_1_ receptors, as described in AERD nasal biopsies [17]. 

This study therefore explored whether IL-13 can upregulate LTC_4_ synthase transcriptional expression in macrophages isolated from resected human lung and whether it leads to a higher release of cys-LTs, either spontaneously or in response to a calcium ionophore, compared with macrophages not cultured with IL-13. Taqman gene expression assays failed to show significant changes in LTC4S mRNA, standardised to the beta-actin housekeeping gene (ACTB), in IL-13 cultures of macrophages from twelve lung tissue donors (Figure 1). Increases in LTC4S mRNA are detectable after six hours of IL-4 treatment in human mast cells derived from cord blood mononuclear cells, and maximal at 24 to 120 hours [12], suggesting that the 16-hour culture with IL-13 employed in our experiments was reasonable. IL-13 may be less potent on myeloid cells than IL-4, and our cells were mature lung macrophages from mainly elderly subjects undergoing lobectomy or bullectomy; these cells may not be as responsive to IL-13 or other stimuli as cord-blood-derived mononuclear cells from healthy neonates. 

Despite the lack of effect on LTC4S transcription, culture of lung macrophages with IL-13 did cause significant increases in cys-LT release, an effect that was apparent both on the low levels of spontaneous cys-LT production in unstimulated cells and on the tenfold higher levels of cys-LT release measured in cells stimulated with calcimycin A23187 (Figure 2). Calcimycin acts as a receptor-independent trigger of arachidonate release from membrane phospholipids; a high turnover of substrate through the 5-LO/LTC_4_ synthase pathway was intended to simulate rate-limiting conditions, such that an increase in LTC_4_ synthase enzyme expression induced by IL-13 could be revealed by a raised ceiling of cys-LT synthesis. In the event, the lack of upregulation of LTC4S mRNA suggests either a nontranscriptional effect of IL-13 or an action on other components of the pathway, possibly on 5-LO activating protein (FLAP), which is inducible by cytokines and increases markedly during human alveolar macrophage maturation [18]. The increases in cys-LT release seen in the IL-13-cultured macrophages were relatively modest (42–52%), but suggest that IL-13 could contribute to increased cys-LT releasability in macrophages in the allergic asthmatic or AERD lung. 

NSAIDs such as indomethacin are proposed to cause acute AERD reactions by shunting of arachidonate from the inhibited COX pathway to the 5-LO/LTC_4_ synthase pathway or by suppressing synthesis of an inhibitory prostanoid such as PGE_2_, thus enhancing cys-LT production [6]. Although isolated anomalies in COX isozyme expression, prostanoid synthesis, and EP receptor signalling have been described in AERD cells and tissues [19, 20], a coherent picture of a systemic prostanoid defect in AERD has yet to emerge [2]. Human alveolar macrophages constitutively express COX-1 and synthesise PGE_2_ [21]. We postulated that endogenous COX pathways in human lung macrophages may therefore provide an adequate target for NSAID action, resulting in enhanced cys-LT release if the 5-LO/LTC_4_ synthase pathway has previously been induced by IL-13. No effect of indomethacin was observed however on either spontaneous or A23187-stimulated cys-LT release in either IL-13-treated or untreated cells (Figure 2). In the absence of an effect of IL-13 on LTC4S mRNA levels, it is not possible to reject our hypothesis that LTC_4_ synthase overexpression in a single cell type could provide a simple cellular model of the key functional changes within the AERD lung. *In vivo*, intercellular interactions may be required, possibly with PGE_2_ derived from airway epithelial cells [22] or it may depend on the recruitment of new populations of LTC_4_-synthase-expressing cells, such as eosinophils. It is nevertheless intriguing that human mast cells derived in culture from the blood mononuclear cells of AERD subjects show an intrinsically raised capacity for cys-LT synthesis and that this is suppressed by PGE_2_ [23], suggesting that the AERD paradigm can be detected at the level of a single cell type and that it persists in prolonged cell culture. Further comparative studies are required in primary lung cell populations from normal and AERD subjects.

The key features of AERD have recently been replicated in ovalbumin-sensitised LTC_4_ synthase transgenic mice (LTC4S-Tg), including its overexpression in airway macrophages and other leukocytes, leading to increased cys-LT synthesis both before and after NSAID challenge, accompanied by dramatically increased Th2 cytokines, including IL-13 [10]. This model suggests that primary dysregulation of LTC_4_ synthase in resident lung cells including macrophages may initiate overproduction of cys-LTs in patients with AERD. The cys-LTS may then promote the secondary synthesis of IL-4, IL-5, and IL-13 from lymphocytes [10] and eosinophils [24], leading to myocyte CysLT1R expression [16], suppression of epithelial PGE_2_ synthesis and EP2 expression [22], and further induction of LTC_4_ synthase [11]. While the LTC4S −444A/C promoter polymorphism [25] has been discounted as an aetiological factor in most AERD populations [26], other genetic, immunological, and microbial factors that could directly dysregulate LTC_4_ synthase in human lung cells merit further investigation.
